# The fallopian tube microbiome: implications for reproductive health

**DOI:** 10.18632/oncotarget.25059

**Published:** 2018-04-20

**Authors:** Elise S. Pelzer, Dana Willner, Melissa Buttini, Louise M. Hafner, Christina Theodoropoulos, Flavia Huygens

**Affiliations:** ^1^ Institute of Health and Biomedical Innovation, School of Biomedical Sciences, Queensland University of Technology, Brisbane, Queensland, 4001 Australia; ^2^ The Wesley Research Institute, Women's Health Laboratory, The Wesley Hospital, Auchenflower, Queensland, 4066 Australia; ^3^ The Australian Centre for Ecogenomics, St Lucia, Queensland, 4067 Australia; ^4^ The Wesley Hospital, Auchenflower, Queensland, 4066 Australia

**Keywords:** fallopian tube, microbiome, menstrual cycle

## Abstract

**Objective:**

There is a paucity of data characterizing the microbiota of the female upper genital tract, which controversially is described as a sterile site. We examine whether the fallopian tube harbours an endogenous microbial community.

**Design:**

This prospective study collected from women undergoing total hysterectomy or salpingectomy-oophorectomy.

**Setting:**

Private hospital gynaecology department.

**Patients:**

Fallopian tubes were collected from women diagnosed with benign disease or for prophylaxis.

**Interventions:**

Samples were interrogated for the presence of microbial DNA using a next generation sequencing technology approach to exploit the V5 to V9 regions of the 16S rRNA gene.

**Main outcome measures:**

The fallopian tube microbiota was characterized using traditional culture techniques and next generation sequencing.

**Results:**

Bacteria were isolated from 50% of cultured samples, and 100% of samples returned positive PCR results. Only 68% of the culture isolates could be confidently identified using automated diagnostic equipment in a clinical microbiology laboratory. Monomicrobial communities were identified only for cultured isolates (50%). Pyrosequencing revealed that all communities were polymicrobial. *Lactobacillus* spp. were not present in all groups, nor were they the most dominant isolates. Distinct differences in the microbial communities were evident for left compared to right fallopian tubes, ampulla versus isthmus, pre- and post- menopausal tissue, and in secretory phase fallopian tubes with and without Mirena intrauterine devices *in situ* (all *p* < 0.05).

**Conclusion:**

The female upper genital tract is not sterile. Distinct microbial community profiles in the fallopian tubes of healthy women suggest that this genital tract site supports an endogenous microbiota.

## INTRODUCTION

Several sexually transmitted microorganisms, including *Chlamydia trachomatis, Neisseria gonorrhoeae* and *Mycoplasma* species have been shown to cause anatomical damage to the human fallopian tube mucosa [1–3]. Microbiological analyses of upper genital tract infections using cultivation have largely focused on these sexually transmitted pathogens, or on organisms known to be associated with bacterial vaginosis. Microbial communities in healthy human fallopian tubes have yet to be well described, as in the absence of disease, they are considered to be sterile. We hypothesized that the healthy fallopian tube harbours a diverse resident microbiota at all stages of the reproductive cycle, even in the absence of infection or inflammation. Here, we use culture-independent microbial community profiling in conjunction with cultivation to characterise this healthy fallopian tube microbiota, explore community-level shifts in microbiota related to hormonal changes and anatomical differences, and determine the pathogenic potential of resident microbial populations. We report striking differences in the microbial diversity of the fallopian tube identified only by the cultivation-independent technique.

## RESULTS

### Patient characteristics and clinical data

The age of the patients ranged from 34–63 years. The cohort included eight pre-menopausal women and eight post-menopausal women. Six women were prescribed oral tinidazole on the evening prior to surgery. All women received IV cephazolin at the time of anaesthetic induction. Histological assessment revealed no remarkable features or evidence of inflammation in any of the fallopian tube samples.

### Primary culture

Bacteria were cultivated from 15/29 (52%) (26 fallopian tube samples collected from women admitted for bilateral salpingectomy and 3 fallopian tube samples collected from women admitted for unilateral salpingectomy) of the fallopian tubes sampled in this study (Table 1). Fifteen bacterial species were able to be identified. Of these, 2/15 (13%) belonged to the genera *Enterococcus* sp., 2/15 (13%) to the genera *Propionibacterium* sp. Standard diagnostic procedures were unable to resolve the identity of 5/29 (17%) of the cultivated isolates. Two Gram-positive isolates could not be identified at any level, and a further three isolates were inadequately identified using automated biochemical analyses, such that key phenotypic, antimicrobial resistance patterns or biochemistry refuted the automated identification.

**Table 1:** Summary of bacterial culture of human fallopian tube samples identified using an automated VITEK system

Bacterial identification	Number of times isolated	Monomicrobial	Vitek identification score
No growth	14 samples	N/A	N/A
*B. vulgatus*	1	0	99%
*Clostridium difficile*	1	1	87%^*^ (No distinctive odour)
*Corynebacterium minutissimum*	1	0	93%
*Eggerthella lenta*	1	0	99%
*Enterococcus durans*	1	1	95%
*E. faecalis*	5	4/5	97–99%
*Erysipelothris rhusiopathiae*	1	0	99%^*^ (TSI not supportive)
*Kocuria rosea*	1	0	91%
*Lactobacillus acidophilus*	2	1/2	50–92%^*^ (one isolate VA SENS, second isolate low sensitivity)
*Propionibacterium acnes*	3	0	89–92%
*P*.*granulosum*	2	0	95%
*Staphlyococcus epidermidis*	3	3/3	94–98%
*S. lugdunensis*	1	0	99%
Unidentified GPB	1	0	^*^
Unidentified GPC	1	0	^*^

Abbreviations: VA = Vancomycin, SENS = Sensitive, ^*^ = incorrect identification, GPB = Gram-positive bacilli, GPC = Gram-positive cocci.

### Microbial community profiling

**Figure 1:**
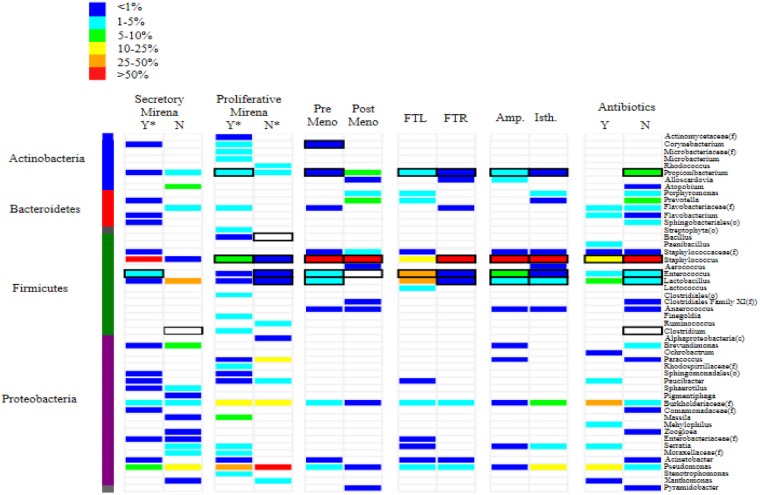
Heat map depicting the most abundant taxa identified in microbial communities found in human fallopian tubes The heat map demonstrates the relative abundance of each community member in the fallopian tube sample. Samples are paired according to menstrual cycle phase, the use of exogenous hormones, anatomical location and pre-surgical antibiotic usage. Each microbial taxon identified for the sample is represented by a coloured bar, the colour of which represents the relative abundance of a given taxon compared to all other taxa identified in that sample. Black rectangles around the coloured bars indicate that specific taxa were also identified from the samples using cultivation-dependent techniques. Y = yes, N = no, meno = menopause, FTL = left fallopian tube, FTR = right fallopian tube, Amp = ampulla, Isth = isthmus, (f) = family, (g) = genus

**Figure 2:**
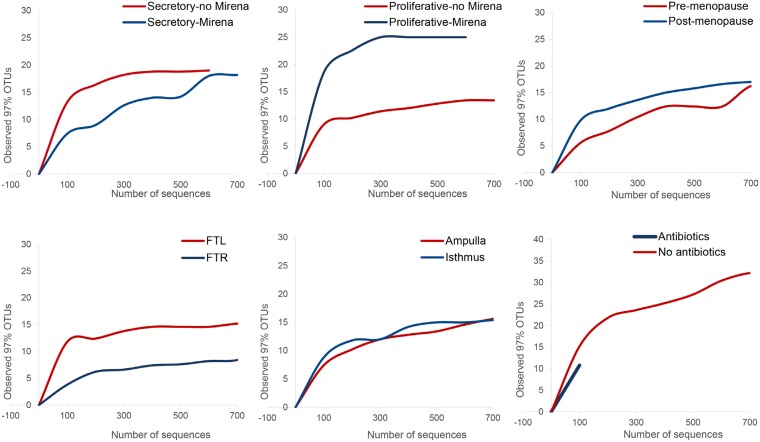
Pairwise comparisons and rarefaction curves for community-level shifts in microbiota related to hormonal changes, anatomical differences and antibiotic usage prior to surgery Rarefaction curves of OTUs clustered at 97% sequence identity across different fallopian samples demonstrate differences in taxonomic richness for cycling women in the presence and absence of exogenous progesterone, for anatomical location and for women who were and were not prescribed prophylactic antimicrobial the night prior to surgery. FTL = left fallopian tube, FTR = right fallopian tube

Microbial community profiling demonstrated that fallopian tubes harbour robust microbial communities which are more diverse than culturing alone would suggest (Figure 1). Rarefaction analysis indicated that the sequencing depth was sufficient to capture the total microbial diversity for most cohorts, and bacterial richness in the fallopian tubes was low compared to other body sites (Figure 2). Microbial communities were dominated by members of the phyla Firmicutes, most notably *Staphylococcus* sp., *Enterococcus* sp., and *Lactobacillus* sp. (Figure 1). Other highly abundant and prevalent taxa included Pseudomonads (*Pseudomonas* sp. and *Burkholderia* sp.) and known genital tract anaerobes such as *Propionibacterium* sp. and *Prevotella* sp.

The results of microbial community profiling were consistent with cultivation for most cohorts, with *Staphylococcus* sp. dominating both the culture-dependent and culture-independent results (Table 2). Notably, neither *Pseudomonas* sp. nor *Burkholderia* sp. were cultured from any cohorts despite their presence in variable abundances in all community profiles (Table 2; Figure 1). In some cases, specific cultured bacteria were not evident in the community profiles: *Enterococcus* sp. in the post-menopausal cohort, and *Clostridium* sp. in women treated with prophylactic antimicrobials prior to surgery, as well as normal cycling women during the secretory phase of the menstrual cycle. This confirms that the suspicious biochemical identification of *Clostridium* sp., with an absence of the characteristic odour was likely inaccurate as indicated by the clinical microbiologist. Additionally, *Bacteroides vulgatus* was not identified in any of the cohorts, but was cultured from normal cycling women during the proliferative phase of the cycle (Table 2). Cultivation-dependent techniques selected for optimal recovery of *Bacteroides* sp. through selective primary isolation media and incubation conditions.

**Table 2:** The most abundant cultivation-dependent and cultivation-independent microbial taxa identified for fallopian tube cohorts defined by anatomy, menstrual cycle phase, menopause and antimicrobial prophylaxis as the time of surgery

Clinical and histological grouping	Cohort	Sample size (*n* =)	Antibiotics^1^ (*n* = )	Culture	454 sequencing^2^
Anatomical site	Left fallopian tube	16	3	*Propionibacterium sp.* *E. faecalis*	*E. faecalis* *Staphylococcus* sp.1 *L. intestinalis* *L. jensenii* *Staphylococcus*. sp.2
	Right fallopian tube	13	4	*Propionibacterium* sp. *E. faecalis Staphylococcus* sp. *Lactobacillus* sp.	*Staphylococcus* sp. *Pseudomonas* sp. *Staphylococcus*. Burkholderiaceae *E. faecalis*
	Ampulla	16	4	*Propionibacterium* sp. *E. faecalis* *Staphylococcus* sp. *Lactobacillus* sp.	*Staphylococcus* sp.1 *E. faecalis* *P. acnes* *Staphylococcus* sp.2 *Staphylococcus* sp.3
	Isthmus	16	4	*Propionibacterium* sp. *E. faecalis* *Staphylococcus* sp. *Lactobacillus* sp.	*Staphylococcus* sp.1 *Pseudomonas* sp. Burkholderiaceae *L. jensenii* *Staphylococcus* sp.2
Menstrual cycle	Mirena	3	1	*E. faecalis*	*Staphylococcus* sp.1 *Pseudomonas* sp. Burkholderiaceae *E. faecalis* *Staphylococcus* sp.2
	No Mirena	1	0	*C. difficile*	*Lactobacillus* sp. *Pseudomonas* sp. *Brevundimonas* sp. *A*.*vaginae* Burkholderiaceae
	Mirena	1	0	*S. epidermidis* *P. granulosum*	*Pseudomonas* sp. Burkholderiaceae *Staphylococcus* sp.1 *Massilia timonae* Flavobacteriaceae
	No Mirena	1	0	*E. faecalis* *B. vulgatus* *L. acidophilus*	*Pseudomonas* sp. *Paracoccus* sp. Burkholderiaceae *Paucibacter* sp. *P. acnes*
Menopause	Pre-menopause	8	3	*P.granulosum* *P. acnes* *E. faecalis* *L. acidophilus* *S. epidermidis* *Corynebacterium* sp.	*Staphylococcus* sp.1 *E. faecalis* *Pseudomonas* sp. *Staphylococcus* sp.2 Burkholderiaceae
	Post-menopause	8	1	*E. durans* *S. epidermidis* *S. lugdunensis*	*Staphylococcus* sp.1 *Staphylococcus* sp.2 *Prevotella* sp. *P. acnes* *Porphyromonas* sp.
Antibiotics	Tinidazole^3^	4	4	*S. epidermidis* *S. lugdunensis*	Burkholderiaceae *Staphylococcus* sp. *Pseudomonas* sp. *L. intestinalis* *Serratia quinivorans*
	No	12	0	*Propionibacterium* sp. *S. epidermidis* *S. lugdunensis* *C. difficile* *Lactobacillus* sp. *E. faecalis*	*Staphylococcus* sp.1. *Staphylococcus* sp.2 *P. acnes* *Prevotella* sp. *Porphyromonas* sp.

^1^ Tinidazole was prescribed to some women in the cohort the night before surgery.

^2^ Top five most abundant microbial community taxa identified by 454 sequencing.

^3^ Prophylactic tinidazole was administered to some women the night prior to surgery.

Multiple members of the *Staphylococcus* family were identified in some cohorts, however, identification to species level was not possible.

### Effects of menstrual cycle and hormones on microbial community composition

Microbial community composition was significantly different between pre- and post- menopausal women, as well as between those receiving exogenous progesterone treatment (Supplementary Table 1). Community composition was compared pairwise between cohorts using Metastats (see Methods) to identify significant differences and the specific taxa driving those differences. Comparative rarefaction analysis was used to explore differences in overall community diversity between pairs of cohorts. *Staphylococcus* sp. was the most abundant bacterial genus recovered from the fallopian tubes throughout all stages of reproductive life in the absence of exogenous hormone treatment (Figure 1). The administration of exogenous progesterone treatment via the use of Mirena intra-uterine devices during the proliferative and secretory phases of the cycle resulted in an increase of staphylococcal dominance (Figure 1; Supplementary Table 1).

Mirena intra-uterine devices also resulted in a significant overabundance of *Pseudomonas* sp., *Brevundimonas* sp. and *Atopobium vaginae* as compared to normal cycling women not exposed to exogenous hormone supplementation during the secretory phase of the menstrual cycle. These women demonstrated an overabundance of *Lactobacillus* sp. and *Enterococcus faecalis* (Supplementary Table 1).

Significant differences in microbial community composition and diversity were also noted between pre- and post-menopausal women. Fallopian tubes from post-menopausal women demonstrated lower taxonomic richness than those collected from pre-menopausal women (Figure 1). In contrast to pre-menopause, in post-menopause there was an absence of lactobacilli and an over-abundance of *Staphylococcus* sp., *Prevotella* sp. and *Propionibacterium* sp. (Supplementary Table 1).

### Biogeography of microbial communities

Microbial communities were compared between the left and right fallopian tubes as well as the ampulla and isthmus to determine if bacterial populations demonstrated site-specific differences. The microbial community within the ampulla demonstrated a significantly greater abundance of *Enterococcus* sp. and *P. acnes* when compared to the isthmus (Supplementary Table 1). Community profiles also differed significantly between the left and the right fallopian tubes. *Lactobacillus* sp., *Enterococcus* sp. and *Prevotella* sp. and were more abundant within the left tube versus the right tube, whilst *Staphylococcus* sp. were more abundant in fallopian tubes collected from the right side.

### Antibiotics and the fallopian tube microbiota

Antibiotic treatment had a significant effect on microbial diversity and community composition (Supplementary Table 1; Figure 2).The antibiotic treated cohort demonstrated lower overall taxonomic richness than the non-treated group (Figure 2). Antibiotic-treatment was associated with the absence of key targeted anaerobes including *Atopobium* sp., *Porphyromonas* sp., *Prevotella* sp. and *Clostridium* sp., which were identified in the corresponding non-treated group. Staphylococci were present in both groups, but were less abundant in the antibiotic-treated group, despite not being a target of the antimicrobial prophylaxis (Supplementary Table 1, Figure 1).

Due to the use of pooled samples to form cohorts, we examined potential confounding factors which might lead to observed differences between the antibiotic-treated and untreated groups. For example, the pre-menopause group was over-represented in the antibiotic treatment group and the post-menopause group was over-represented in the non-treatment group; however, some key distinguishing community members were not detected in the pre-menopausal and post-menopausal cohorts (*Serratia* sp., members of the genera *Flavobacterium, Paenibacillus* and *Paucibacter* and the Comomonadaceae family). Of note, it is possible that these taxa may also be present in the community in numbers below the level of detection.

## DISCUSSION

We have demonstrated using both cultivation and culture-independent microbial community profiling, that in the absence of infection, the human fallopian tube is not a sterile site. Data from the human microbiome project continues to reveal the complexity of microbial communities within the female lower genital tract, highlighting the impact of the bacterial community, the menstrual cycle and sexual activity [4–6]. More recently, low-biomass fallopian tube, endometrial and placental microbiomes have been characterized in the absence of disease [7–9]. Non-cultivable bacteria have recently been isolated from the uninfected adult female bladder, a site previously described as sterile [10]. Isolates were closely related to taxa reported in molecular analyses of the female vagina, further supporting the notion that much of the upper genitourinary tract is not sterile [10, 11]. Previously reported abundant community members are similar to those identified in our current study. Pseudomonads that were not detected by cultivation-dependent techniques were also detected in the sample profiles. Whilst these genera represent common reagent contaminants, as obligate aerobes, they were also selectively excluded from cultivation as a result of incubation conditions commonly employed for genital tract samples. Reagent contamination is more frequently associated with pyrosequencing of samples with a ratio of very low microbial DNA to very high mammalian DNA [12–14]. This is true for some, but not all of our samples. Our results suggest that reagent contamination cannot sufficiently explain the abundance of the pseudomonads across all study groups.

We are cognisant of our small sample size; however, we propose that the dominance of staphylococci we report at this site may underpin reports of staphylococcal-associated tubo-ovarian abscess following tubal ligation [11, 15]. Laparoscopic recovery of *S. epidermidis* from the cul-de-sac, but not the vagina or endocervix from the same patient has previously been reported [16]. Staphylococci have also been recovered from the uterus but not vaginal vault or cervix in women undergoing hysterectomy and in cases of salpingitis, pelvic inflammatory disease and peritonitis [17–20].

This study confirms the impact of hormonal changes throughout the menstrual cycle on the normal resident microbiota. In line with previous reports, post-menopausal women demonstrated an absence of lactobacilli when compared to the pre-menopausal cohort. We also reported alterations as a result of the local administration of exogenous progesterone via an intra-uterine device. Staphylococci have been recovered from intrauterine devices removed from women with genital tract infections, possibly supporting a role for their increased population abundance in the cohorts of women in our study with a Mirena intrauterine device *in situ* [21]. Our results demonstrate an inverse relationship between *Staphylococcus* sp. abundance compared to *Pseudomonas* sp. abundance. A decrease in staphylococcal abundance has previously been reported in the presence of highly abundant pseudomonads [22]. Interestingly, a recent study reported a high abundance of staphylococci in the ovarian cancer oncobiome, for which the fallopian tube may be a seeding site [23].

The observation of differences between the microbial communities in the left and right fallopian tubes and between the ampulla and isthmus is likely related to the differences in vasculature, pelvic anatomy and peritoneal flow [24]. The ampulla is the portion of the fallopian tube most often associated with salpingitis, ectopic pregnancy implantation and tubo-ovarian abscess [25].

Differences within the microbial community profiles of antibiotic treated and non-treated women (tinidazole the night prior to surgery) may represent a community shift resulting in the ability of other community members to dominate. The antimicrobials selected in previous studies are mostly ineffective against the anaerobes and staphylococci identified as the most abundant members of the fallopian tube microbial community in our current study [26, 27].

The moderate-spectrum cephalosporins and the nitroimidazoles are the antimicrobials recommended for prophylaxis in gynaecologic surgery, and with resistance reported against metronidazole, it may be tempting to use tinidazole [26]. However, in a climate where antimicrobial resistance is increasing, it would be prudent to reserve tinidazole treatment for more at-risk patients with a history of bacterial vaginosis. We would suggest that the single-dose cefoxitin, which has some anti-anaerobic activity, is appropriate for the majority of gynaecologic cases, particularly those focused on fallopian tube removal/resection.

Previous studies investigating the relationship between bacteria and fallopian tube pathology have largely assumed sterility at this site [1–3]. However, most prior studies did not screen for the presence of endogenous microbiota or relied on cultivation alone, and thus may have missed low abundance populations or those which do not easily grow in culture. Microorganisms participate in complex intraspecies and interspecies interactions. It is therefore possible that some pathology associated with sexually transmitted pathogens including *Chlamydia* sp. and *Mycoplasma* sp. are actually due to pathogenic synergism between the identified pathogen and non-cultivated members of the endogenous microbial community. Such interactions may explain why not all women with sexually transmitted pelvic infections progress to tubal factor infertility and why the women in this current study had no apparent pathology in the presence of a diverse microbial community in the fallopian tube.

Molecular microbiology analyses of the fallopian tube should be considered in patients where other causes of infertility or adverse outcomes such as ectopic pregnancy, hydrosalpinges or miscarriage, have been discounted. Removal of fallopian tubes in women with hydrosalpinges has resulted in improved outcomes, and based on our data, this may be due to the presence of an endogenous microbial community in the fallopian tube that is interfering with normal reproductive function in some women [28].

Fallopian tubes in asymptomatic women contain detectable and diverse microbial communities, which are affected by hormones and antibiotics, and display biogeographical tropism. In some women there can be an imbalance in this flora - dysbiosis. Further, this pilot study serves as a reminder of the limitations of both molecular and traditional microbiology techniques in characterizing human microbiota. A key limitation of 16S rRNA sequencing is an inability to consistently and confidently discriminate microbial identity beyond family or genus level, evidenced in this current study as *Staphylococcus* spp., which includes potentially all staphylococci including *S. epidermidis*, which we detected using traditional culture techniques but did not identify to species level by 16S rRNA [29–31]. Similarly, cultivation techniques are limited by readily generating growth of non-fastidious species only on solid primary isolation media. Lactic acid bacteria have well-described inherited adaptation mechanisms for survival in suboptimal conditions, frequently entering a dormant or persistent state, which supports our findings of limited recovery of lactobacilli using traditional culture techniques [32, 33]. Larger prospective studies are required to explore the true impact of the fallopian tube microbiota on reproductive health outcomes.

We have shown that in our study population, antimicrobial prophylaxis prior to gynecologic surgery alters the fallopian tube microbiota, resulting in undetectable *Atopobium* sp., *Porphyromonas* sp., *Prevotella* sp. and *Clostridium* sp., as well as reduced detection of staphylococci. Each of these bacterial species are frequently implicated in reproductive tract infections. We also observed alterations in the fallopian tube microbiota for pre-and post-menopausal women, and in response to exogenous hormone treatment. These findings provide important insights into the labile nature of the fallopian tube microbiota, which should be considered as a potential source of microbial seeding in post-surgical infection, and a possible cause of reproductive pathology

## MATERIALS AND METHODS

### Ethics statement

Ethical approval was obtained from the review boards of UnitingCare Health, Human Research Ethics Committee and Queensland University of Technology Human Ethics Committee. All patients provided informed written consent for excess fallopian tube tissue to be used in this study.

### Study subjects and tissue collection

Women undergoing total hysterectomy with salpingectomy or salpingectomy-oophorectomy or laparoscopic salpingectomy or salpingectomy-oophorectomy only for benign disease or prophlyaxis at the Wesley Hospital (Brisbane, Australia), were invited to enroll in this study. Bilateral fallopian tubes were collected from 13 women for testing and unilateral fallopian tubes were collected from the remaining 3 of the 16 women enrolled in this study.

Following excision by the surgeon, fallopian tubes were immediately placed into sterile containers and transported to the pathology department. Pathology staff performed the gross tissue dissection using sterile instruments. Excess tissue not required for diagnostic purposes was immediately placed into cold RPMI media (Sigma Aldrich, Australia) and transported to the microbiology laboratory for processing.

### Primary culture and ASA

In the first instance, 200 μL of each of fallopian tube culture specimen was inoculated into thioglycollate broth (Oxoid, Australia) and incubated at 37° C for 48 hours. A sterile swab (Interpath, Australia) was used to subculture the thioglycollate broths onto a range of culture media (horse blood agar, chocolate I agar, anaerobic blood agar) (Oxoid, Australia) using the 16-streak technique. Plates were incubated aerobically, in 5% CO2 or anaerobically in anaerobic jars (Oxoid anaerogen, 2.5 L, Oxoid, Australia) at 37° C. Colony forming units (CFUs) on plates incubated aerobically were counted after 24 hours, whilst those on plates incubated under CO_2_ were counted after 24 hours and again at 48 hours (Isenberg, 2009). The anaerobic plates and thioglycollate broths were examined and CFUs enumerated at 48 hours and then every second day up to 14 days. Each different colony type from all plates was Gram-stained and subcultured for biochemical identification.

Identification of clinical isolates was performed on the VITEK 2 (Biomerieux, France) using the appropriate identification cards (Gram-positive GP, Corynebacteria CBC or Anaerobe and Coryneform ANC) according to manufacturer's instructions. With a vacuum device, a homogenous suspension of the isolate taken from non-selective media was prepared to a specified McFarland standard. The cards were automatically sealed and then inserted into the reader inoculator module of the VITEK 2. Fluorescence was measured at 15-minute intervals until identification was determined.

Antimicrobial susceptibility assays were performed with the VITEK 2 for isolates using the AST cards where appropriate (Biomerieux, France), according to manufacturer's instructions. The antimicrobial agents included on the card were as follows: Ampicillin, Cefoxitin Screen, Ciprofloxacin, Clindamycin, Daptomycin, Doxycycline, Erythromycin, Gentamicin, Levofloxacin, Linezolid, Moxifloxacin, Nitrofurantoin, Oxacillin, Rifampicin, Streptomycin, Tetracycline, Tigecycline, Trimethoprim/Sulfamethoxazole and Vancomycin. The cards were filled with an inoculum of the manufacturer's specified density and then sealed and read. The system automatically processed the cards until the minimum inhibitory concentrations (MICs) were obtained.

### Fallopian tube cohorts

Fallopian tube cohorts were constructed by collating the clinical history and histopathology reports from each woman, to consider factors capable of influencing the microbial community such as anatomy, menstrual cycle phase, menopause and antimicrobial prophylaxis (Table 1). We analysed data from four anatomical cohorts: left fallopian tubes (*n* = 16), right fallopian tubes (*n* = 13), ampulla (*n* = 16) and isthmus (*n* = 16). We examined samples collected from women during the proliferative and secretory phases of the menstrual cycle with and without Mirena intrauterine devices (*n* = 1, 1, 1 and 3 respectively), from pre-menopausal (*n* = 8) and post-menopausal (*n* = 8) women and from women with and without antimicrobial prophylaxis the night prior to their surgical procedure (*n* = 4 and 12 respectively). Each cohort was constructed by pooling an equal concentration of DNA extracted from each individual sample.

### DNA extraction

DNA extraction was performed on 1 mL aliquots of each fallopian tube in culture media, and the extracted DNA was used for 16S rDNA PCR (described below). The culture medium from each fallopian tube section was centrifuged at high speed and the pellet was resuspended in lysis enzyme. The suspension was vortexed and incubated at 37° C for one hour. DNA was extracted using a Qiagen QiAMP Mini DNA extraction kit (Qiagen, Australia) as per the manufacturer‘s instruction. A culture medium only extraction was included as a negative control. The final DNA elution was into 50 μL of sterile distilled water.

### 16S rDNA PCR

*In silico* PCR performed to select the best target region for the genital tract, based on data available for vaginal samples, well-accepted variability in 16S rRNA primer pair to amplify all species equally [34]. The 16S rDNA PCR assay was performed using the previously published primers, 803F 3′- GATTAGAT ACCCTGGTAG-5′ and 1392wR 5′-ACGGGCGGTG TGTRC-3′ and PCR cycling conditions (Willner *et al.*, 2012). This primer pair was also optimised to minimize amplification of mammalian DNA in what was expected to be low biomass sample. The PCR master mix included: 1 × buffer; 200 μM of dNTPs (Roche, Australia); 0.5 μM of each primer (Sigma Aldrich, Australia); 5 U *Taq* polymerase (Roche, Australia) and 8 μL of the extracted DNA to a final volume of 50 μL. PCR cycling conditions included: an initial denaturation at 95° C for 5 minutes; followed by 30 cycles of denaturation at 95° C for 30 seconds, primer annealing at 55° C for 45 seconds, extension at 72° C for 90 seconds; and a final extension step at 72° C for 10 minutes (PTC-200, Peltier Thermal Cycler, BioRad, Australia).

### Microbial community profiling

Fusion primers with 454 adaptor sequences were ligated to the previously published primers: 803F 3′- GATTAGATACCCTGGTAG-5′ and 1392wR 5′-ACGGGCGGTGTGTRC-3′ to amplify the V5 to V8 regions of the 16 S rRNA gene [35]. PCR reactions were performed in 50 μL reaction mixtures containing: 5 μL of template DNA (PCR product), 5 μL of 10× buffer (Invitrogen, USA), 1 μL of 10 mM dNTP mix (Invitrogen, USA), 1.5 μL BSAI (Fermentas, USA), 1.5 μL 50 mM MgCl_2_, 1 μL of each 10 μM primer, and 1 unit of Taq polymerase (Invitrogen, USA). Cycling conditions included: an initial denaturation at 95° C for 3 minutes, followed by 30 cycles of denaturation at 95° C for 30 seconds, primer annealing at 55° C for 45 seconds and extension at 72° C for 90 seconds followed by a final elongation at 72° C for 10 minutes. Following amplification, PCR products were purified using the QIAquick PCR purification kit (Qiagen, Australia) as per manufacturer's instructions and sequenced using the 454 GS-FLX Titanium platform.

### Bioinformatics and biostatistics

Amplicon sequences were length filtered and quality trimmed using Acacia, which also corrects homopolymer errors [36]. Sequences were assigned to their respective samples based on oligonucleotide barcodes using QIIME. Sequence clustering and operational taxonomic unit (OTU) selection was performed using a modified version of CD-HIT-OTU-454 which does not remove singleton clusters [37]. Taxonomy was assigned to representative sequences by comparison to the latest build of the Greengenes database using BLAST, and OTU tables were constructed from the output using a custom Perl script [38]. Genus-level OTU tables were generated using QIIME, and were visualised using the function heatmap.2 in the R gplots library [39]. Comparisons between paired samples were performed using the non-parametric statistical software Metastats which corrects for multiple comparisons is robust for sparse data [40].

## SUPPLEMENTARY MATERIALS TABLE


