# Supplementary material for: The fallopian tube microbiome: implications for reproductive health
Source: Oncotarget. 2018 Apr 20;9(30):21541–51. doi: 10.18632/oncotarget.25059 (PMC5940370; doi:10.18632/oncotarget.25059)
Supplement: Supplementary file 1 [file oncotarget-09-21541-s001.pdf]

The fallopian tube microbiome: implications for reproductive health

SUPPLEMENTARY MATERIALS

Supplementary Table 1: Changing community abundance in response to menstrual cycle hormones

Over abundant microbial taxon	Secretory-no Mirena	Secretory-Mirena	Pre-meno	Post-meno	FTL	FTR	Ampulla	Isthmus	Antibiotics	No antibiotics
A. vaginae	***									
Brevundimonas	***									
Burkholderiaceae								***	***	
Comomonadaceae (family)										***
E. faecalis		***			***		***			
Flavobacterium (genus)									***	
Lactobacillus	***				***					
P. acnes				***			***			***
Paenibacillus (gen)									***	
Paucibacter (gen)									***	
Prevotella 1 (gen)				***	***					***
Prevotella 2										***
Pseudomonadaceae (fam)									***	
Pseudomonas	***							***		
Serratia quinivorans									***	
Staphylococcus		***		***		***				***

*** = $P < 0.0001$.
